# Reduced graphene oxide-supported Ag-loaded Fe-doped TiO_2_ for the degradation mechanism of methylene blue and its electrochemical properties†

**DOI:** 10.1039/c7ra13418e

**Published:** 2018-02-09

**Authors:** Dhayanantha Prabu Jaihindh, Ching-Cheng Chen, Yen-Pei Fu

**Affiliations:** Department of Materials Science and Engineering, National Dong Hwa University Shou-Feng Hualien 97401 Taiwan ypfu@gms.ndhu.edu.tw

## Abstract

Graphene oxide-based composites have been developed as cheap and effective photocatalysts for dye degradation and water splitting applications. Herein, we report reduced graphene oxide (rGO)/Ag/Fe-doped TiO_2_ that has been successfully prepared using a simple process. The resulting composites were characterized by a wide range of physicochemical techniques. The photocatalytic activities of the composite materials were studied under visible light supplied by a 35 W Xe arc lamp. The rGO/Ag/Fe-doped TiO_2_ composite demonstrated excellent degradation of methylene blue (MB) in 150 min and 4-nitrophenol (4-NP) in 210 min under visible light irradiation, and trapping experiments were carried out to explain the mechanism of photocatalytic activity. Moreover, electrochemical studies were carried out to demonstrate the oxygen evolution reaction (OER) activity on rGO/Ag/Fe-doped TiO_2_ in 1 M of H_2_SO_4_ electrolyte, with a scan rate of 50 mV s^−1^. The reductions in overpotential are due to the d-orbital splitting in Fe-doped TiO_2_ and rGO as an electron collector and transporter.

## Abstract 2

Graphene oxide-based composites have been developed as cheap and effective photocatalysts for dye degradation and water splitting applications.

## Graphical abstract

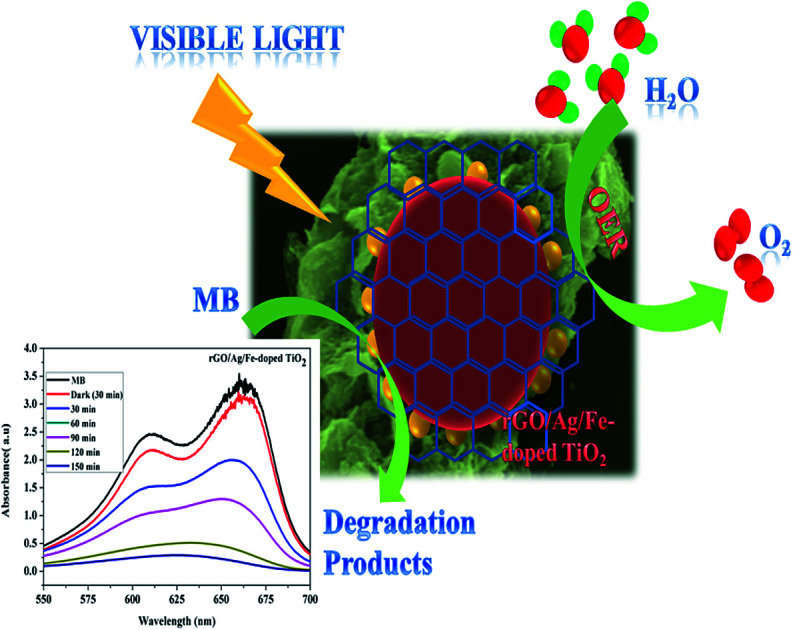

## 1. Introduction

In recent years, the increasing release of dye wastewater from various industries, such as textiles, printing, food, and cosmetics, has become a major threat to humans and ecology owing to the toxicity and non-biodegradability of dye wastewater.^1,2^ Industrially exuded wastewater contains many harmful contaminants such as heavy metals and dyes, which are considered carcinogenic.^3–7^ Moreover, the need for clean and renewable energy has inspired researchers to investigate semiconductors, such as TiO_2_, as photocatalysts for water splitting, the oxygen evolution reaction (OER), the oxygen reduction reaction (ORR), as well as environmental clean-up because industrial waste often contains notable concentrations of synthetic organic dyes.^8–10^ The large band gap of TiO_2_ restricts its practical applications under visible-light illumination, which covers a wide range of the solar spectrum. To overcome this problem, several attempts have been made such as (1) doping a transition metal ion into an anatase TiO_2_ lattice; in this regard, Fe^3+^ ions have attracted significant attention due to their half-filled d-electronic configuration and identical ionic radius to Ti^4+^; this implies that the Fe^3+^ ion may be incorporated into the structure of the TiO_2_ lattice.^11^ Moreover, doping of Fe^3+^ ions reduces the recombination of the photogenerated electrons and holes; this leads to the narrowing of the band gap energy of TiO_2_.^12–15^ (2) Depositing noble metal nanoparticles, such as Ag nanoparticles, as electron-transfer co-catalysts, which have a broad range of visible spectra absorption as well as strong localized surface plasmon resonance (LSPR).^16^ (3) Graphene composites and TiO_2_ nanoparticles have the advantages of increasing the absorptivity of pollutants, extended light absorption range, and facile charge transportation and separation.^17,18^

Graphene, a two-dimensional layer of sp^2^-hybridized carbon atoms, has been widely used in sensors, electronics, drug delivery, supercapacitors, and catalysis due to its unique electrical properties,^19,20^ high thermal conductivity,^21^ mechanical strength, and specific surface area.^22^ However, graphene has some limitations for wide use in wastewater treatment due to its water-insoluble properties.^23^ Recently, several studies have contributed to the investigation of the catalytic or photocatalytic properties of graphene or reduced graphene oxide (RGO).^17,24–26^ The oxidized derivative of graphene, such as graphene oxide (GO), contains various functional groups such as carboxyl, hydroxyl and epoxide on its surface, which makes it highly hydrophilic and water soluble. This makes it applicable for supporting metal/metal oxide particles.^27^ Compared with GO, reduced graphene oxide (rGO) has higher electrical conductivity and thermal stability, which may promote electron transport speed in the Fenton process.^28,29^ Also, these rGO/TiO_2_-based composites have been used in many applications such as photocatalysis, solar cells, and hydrogen evolution.^30–33^ TiO_2_-based photocatalysts are poorly used in oxygen evolution reactions (OER). To date, expensive noble metals such as Pt, RuO_2_ and IrO_2_ electrocatalysts are widely used because of their excellent characteristics.^34,35^ These include low over-potential, excellent reaction kinetics and an outstanding long-term durability in acidic media for the oxygen reduction reaction (ORR), which is important for developing new noble metal free electrocatalysts and exhibiting good electrochemical activity and stability in acidic operating conditions.^36^

In the present study, we report a Ag loaded Fe-doped TiO_2_ on rGO (rGO/Ag/Fe-doped TiO_2_) synthesized for the removal of toxic methylene blue (MB) dye from wastewater, and OER in the acidic electrolyte. During the *in situ* synthesis of GO/metal oxide nanocomposites, GO was reduced to rGO. The rGO/Ag/Fe-doped TiO_2_ composite consists of unique properties for each constituent; *e.g.*, TiO_2_ particles degrade organic pollutants, Fe^3+^ acts as an electro-transfer cocatalyst and rGO provides an effective pathway to increase the surface area as well as suppress the recombination of charge carriers in TiO_2_. Ag nanoparticles also act as electro-transfer cocatalysts and active reaction sites on the graphene surface to improve the interfacial catalytic performance. Herein we report MB and 4-nitrophenol degradation, and photoelectrochemical properties of photocatalysts, such as TiO_2_, Fe-doped TiO_2_, rGO/Fe-doped TiO_2_ and rGO/Ag/Fe-doped TiO_2_, using a 35 W Xe arc lamp.

## 2. Experimental

### 2.1. Materials

All the chemicals used in the study were analytical grade. Titanium dioxide powders (TiO_2_), ferric oxide (Fe_2_O_3_), sulfuric acid (H_2_SO_4_), and sodium nitrate (NaNO_3_) were purchased from Shimakyu's Pure Chemicals, Japan. Potassium permanganate (KMnO_4_) and hydrogen peroxide (H_2_O_2_, 30 vol%) were purchased from Choneye Pure Chemicals, China, and 4-nitrophenol was purchased from Acros Organics, U.S.A. All chemicals were used without further purification.

### 2.2. Preparation of rGO/Ag/Fe-doped TiO_2_

**Fig. 1:**
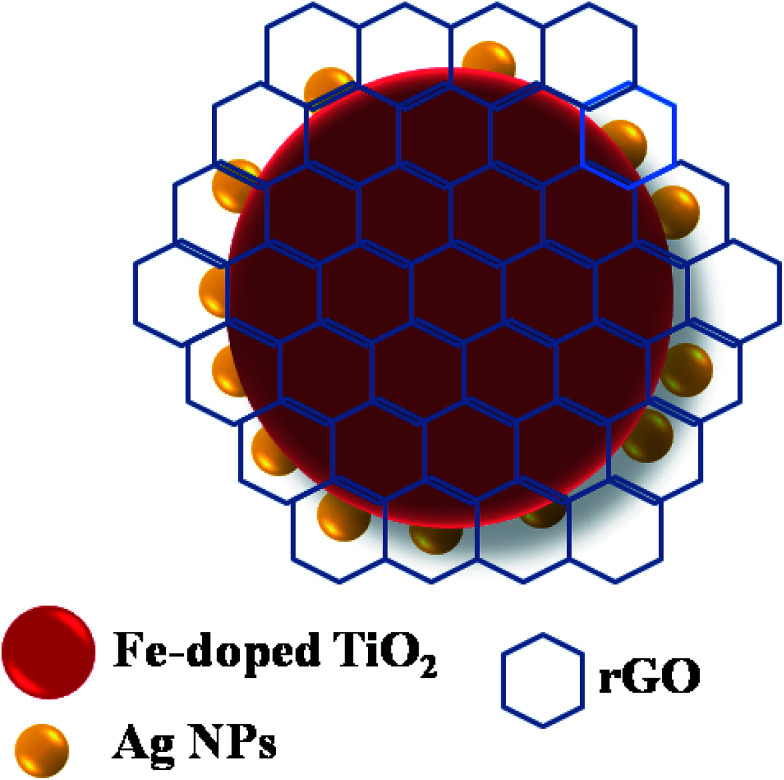
Schematic of the rGO/Ag/Fe-doped TiO_2_ composite.

The synthesis of Fe-doped TiO_2_ has been elaborated on in a previous contribution, and GO was prepared from graphite powder according to a modified Hummers' method.^37–41^ Briefly, the Ag-loaded Fe-doped TiO_2_ and rGO/Ag/Fe-doped TiO_2_ were prepared by a simple chemical reduction and the hydrothermal method as follows. Fe-doped TiO_2_ (0.4 g of 2 mol%) was sonicated with 20 mL of deionized water for 10 min to get a good dispersion of materials and then water was removed by centrifugation. In another beaker, 0.1 M of AgNO_3_ (1 wt% of Ag) and 5 M of NH_4_OH were dissolved in 10 mL of DI water and then Fe-doped TiO_2_ powder was added to the silver nitrate solution, the mixture was sonicated for 10 min and then 0.03 g mL^−1^ of glycerin was added to reduce AgNO_3_ to Ag nanoparticles. The solution was stirred for 3 h at room temperature. The final product obtained was washed with ethanol a few times and dried in a hot air oven at 70 °C for 24 h. The rGO/Ag/Fe-doped TiO_2_ composite was prepared as follows. First, 5 wt% (0.025 g) of GO was taken with 20 mL of ethanol and was ultrasonicated to get a better dispersion and then 0.5 g of Ag loaded Fe-doped TiO_2_ powder was added followed by 10 mL of DI water. This mixture was stirred for 15 min to obtain a homogeneous solution, which was then transferred into a Teflon sealed autoclave and heated at 120 °C for 12 h. Afterwards, the precipitate was washed with DI water a few times and dried in an oven at 70 °C for 24 h. Finally, the hydrothermally reduced graphene oxide was connected to the Ag loaded Fe-doped TiO_2_ (Fig. 1).

### 2.3. Characterization

A computerized X-ray powder diffractometer (XRD) with Cu Kα radiation (*λ* = 0.15406 nm) (Rigaku D/Max-II) was used to identify the GO, rGO, rGO/Fe-doped TiO_2_ and rGO/Ag/Fe-doped TiO_2_. Their particle-size, morphology, and composition were observed and analyzed using a scanning electron microscopy (SEM; Hitachi 3400N) equipped with an energy dispersive spectrometer (EDS). Electron probe microanalysis (EPMA) was employed to study the elements on the rGO/Ag/Fe-doped TiO_2_ composite using an electron probe X-ray microanalyzer (JEOL JXA-8200). The particle size, shape and selected area electron diffraction (SAED) pattern were observed by transmission electron microscopy (TEM; JEOL JEM-3010, Tokyo, Japan). X-ray photoelectron spectroscopy (XPS; VGS Thermo K-Alpha) with an Al Kα radiation as the exciting source was adapted to assist us in figuring out the chemical composition. All the binding energies referenced the C 1s peak at 284.3 eV. The UV-vis spectra were obtained using a UV-vis recording spectrophotometer with an integrating sphere (Jasco, V-600). Photoluminescence spectra were measured using a PerkinElmer LS 45 with Xe lamp. The properties of GO, rGO, and other TiO_2_-based photocatalysts were characterized by micro-Raman (Renishaw, 1000B). Fourier transform infrared (FTIR) spectroscopy studies were carried out using a PerkinElmer Spectrum. Samples for analysis were prepared by the KBr pellet method. The weight ratio of sample/KBr was 5 : 100.

### 2.4. Photocatalytic activity

The catalytic reaction for the degradation of aqueous methylene blue (MB) was carried out in a 200 mL Pyrex glass vessel with magnetic stirring. The initial concentration of the methylene blue was set to 20 mg L^−1^ (20 ppm). The photocatalyst (10 mg) was mixed with 50 mL of MB solution. The photocatalytic activities were evaluated by measuring the photocatalytic degradation of MB using a 35 W Xe arc lamp and color temperature of 6000 K as the visible-light source. Illumination was implemented after the suspension was ultrasonicated for 10 min, then it was left in a dark room for 20 min to reach adsorption–desorption equilibrium. At specific time intervals (every 30 min), 5 mL of the sample was taken from the suspensions and centrifuged to remove photocatalyst particles prior to spectral measurement. MB was monitored by measuring the absorbance at a wavelength of 664 nm, characteristic of MB. To understand the mechanism of photocatalytic degradation, the trapping experiment was carried out by using different kinds of scavengers such as isopropyl alcohol (IPA) as the hydroxyl radical scavenger and formic acid (CH_2_O_2_) as the holes scavenger and the addition of tetrachloromethane (CCl_4_) and *para*-benzoquinone (PBQ) as the electron and superoxide radical scavenger. The scavenger concentrations were set at 1 mmol for the trapping experiment. The same procedure was followed for 4-NP degradation; the initial concentration of 4-NP was set to 20 mg L^−1^ (20 ppm), and 10 mg of photocatalyst was mixed with 50 mL of 4-NP solution. The adsorption of 4-NP was evaluated in the dark and it was found negligible after 1 h for all of the photocatalysts. 4-NP was monitored by measuring the absorbance at a wavelength of 315 nm characteristic of 4-NP.

### 2.5. Photoelectrochemical properties

Photoelectrochemical properties was determined by using a three electrode cell consisting of a working electrode (WE), Pt as the counter electrode (CE), and Ag/AgCl (in 3 M KCl) as the reference electrode (RE). H_2_SO_4_ solution (1 M) was used as an electrolyte. The electrochemical measurements were performed using a potentiostat/galvanostat (CHI, 6273D) at room temperature. The catalysts inks were prepared by ultrasonication of a turbid solution containing 20 mg of photocatalytic materials with 300 μL of deionized water and 30 μL of 5% Nafion for 20 min. A known amount of the catalyst ink was taken and placed on a glassy carbon electrode (GCE) with an active surface area of 0.071 cm^2^, which acted as the working electrode in the three electrode cell system. The oxygen evolution reaction (OER) was carried out using a 35 W Xe arc lamp with color temperature 6000 K and emissions in the range of 360–1000 nm were used to irradiate the samples.

## 3. Results and discussion

### 3.1. Composition and morphology characterization

**Fig. 2:**
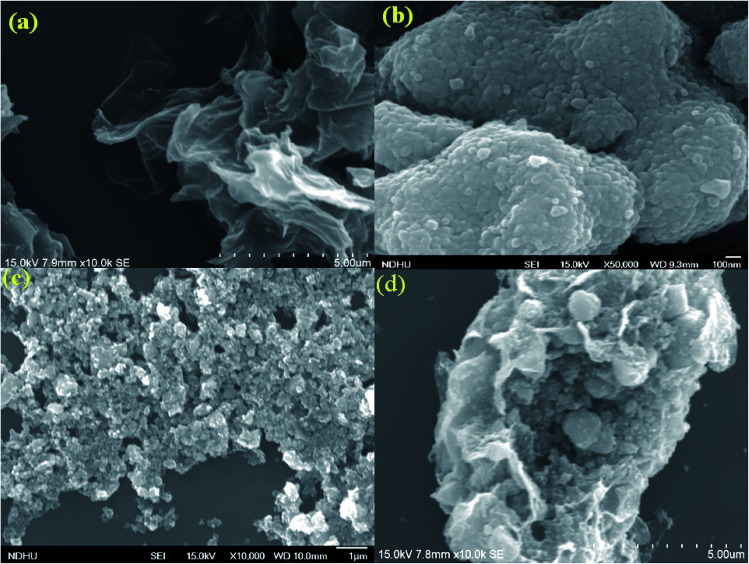
SEM characterization of (a) rGO, (b) TiO_2_, (c) Fe-doped TiO_2_, and (d) rGO/Ag/Fe-doped TiO_2_.

**Fig. 3:**
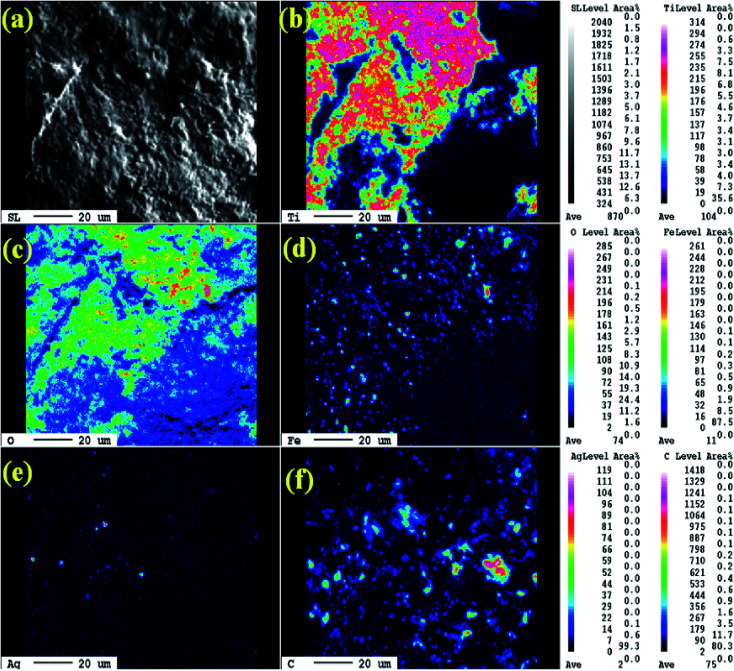
(a) SEM images of rGO/Ag/Fe-doped TiO_2_ and the EPMA elemental mapping of (b) Ti, (c) O, (d) Fe, (e) Ag, and (f) C.

**Fig. 4:**
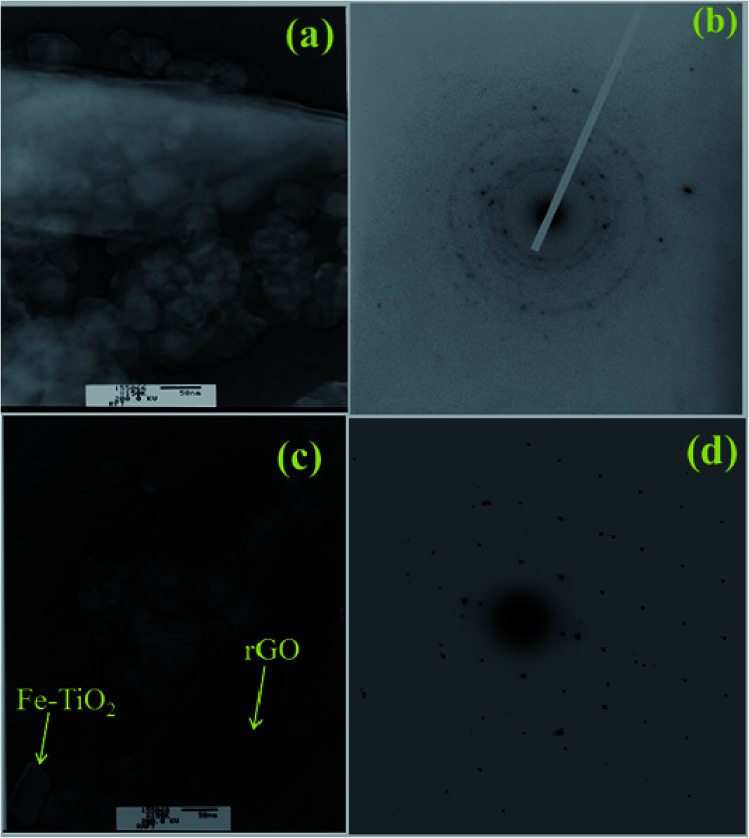
TEM characterization of (a) rGO/Fe-doped TiO_2_ and (c) rGO/Ag/Fe-doped TiO_2_. (b) Electron diffraction patterns of rGO/Fe-doped TiO_2_ and (d) rGO/Ag/Fe-doped TiO_2_.

The morphology and grain size of TiO_2_, Fe-doped-TiO_2_, rGO and rGO/Ag/Fe-doped TiO_2_ composite were investigated by SEM. The SEM image of rGO in Fig. 2(a) reveals a platelet-like interlinked structure. Fig. 2(b) depicts agglomerated TiO_2_ particles with the individual grain size of around 100 nm. Fig. 2(c) shows the Fe-doped TiO_2_, which shows that there is no significant difference between TiO_2_ and Fe-doped TiO_2_, because the Fe ions were totally incorporated into the crystal structures of TiO_2_. Fig. 2(d) shows the rGO/Ag/Fe-doped TiO_2_ composite; the Ag/Fe-doped TiO_2_ particles were well wrapped and connected to reduced graphene oxide. The good connection between Ag/Fe-doped TiO_2_ and rGO therefore facilitated the transfer of photo-induced electrons during the photoexcitation process, which enhanced photocatalytic activity of the composite. To further identify the element distribution in the rGO/Ag/Fe-doped TiO_2_ composite, EPMA was employed to characterize Ti, O, Fe, Ag, and C elements in the specimens. Fig. 3(a) shows the rGO/Ag/Fe-doped TiO_2_ image, which was pressurized into a tablet for EPMA analysis and led to the agglomeration of the composite. Fig. 3(b) and (c) show the element mapping for Ti and O, which were homogeneously distributed. Clearly, there was interaction between Ti and O due to the formation of TiO_2_. Fig. 3(d) and (e) show the element mapping for Fe and Ag; the contents of Fe and Ag were significantly lower than Ti and O, as was expected. The lowest element content was C from rGO, which was ascribed to the fact that carbon is very light and is not easy to detect accurately and therefore the C distribution amount may not be a real reflection in the mapping image. The colored scale bar shows the relative concentration of elements in the scanned area (20 μm). In the colored scale bar, the relative concentration of elements increased from blue to red and the elemental count levels, area%, are shown in the right side of the EPMA images. Fig. 4(a) and (b) show the TEM images for rGO/Fe-doped TiO_2_ and rGO/Ag/Fe-doped TiO_2_, respectively. The corresponding electron diffraction patterns of rGO/Fe-doped TiO_2_ and rGO/Ag/Fe-doped TiO_2_ are shown in Fig. 4(b) and (d), respectively. The rings in Fig. 4(b) correspond to Fe-doped TiO_2_ and the electron diffraction in Fig. 4(d) shows a typical octahedral geometry, which is in good correspondence with Ag and Fe-doped TiO_2_ polycrystalline nanopowders.^42^

**Fig. 5:**
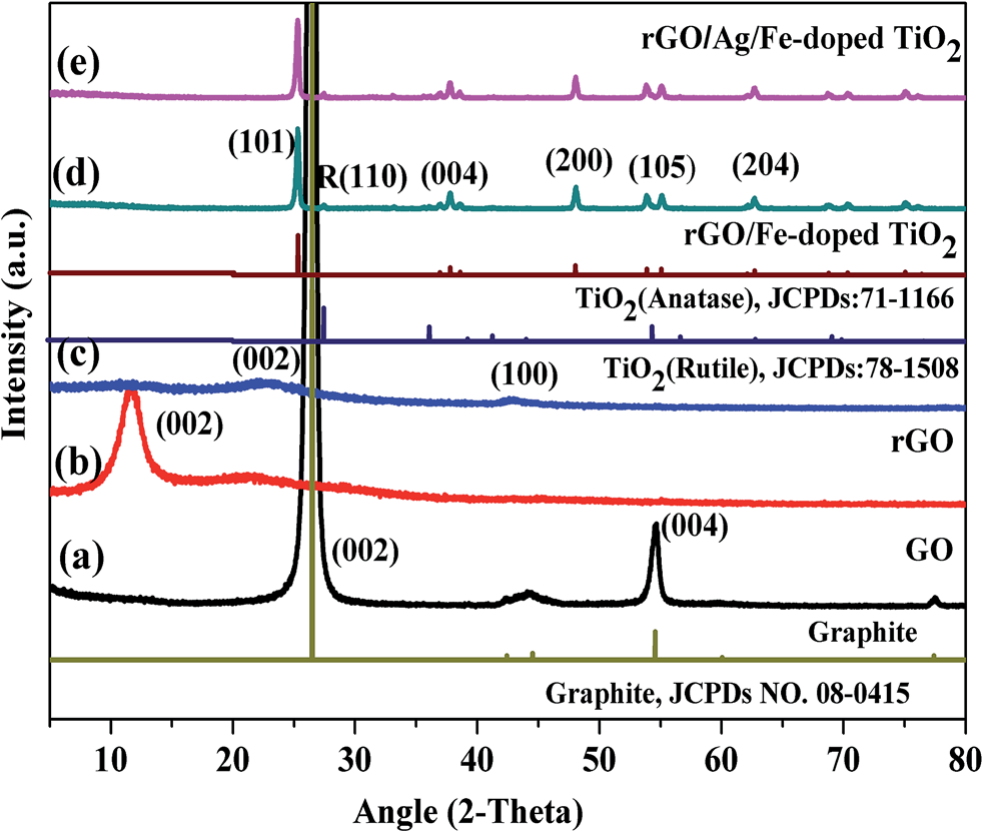
XRD patterns of (a) graphite, (b) GO, (c) rGO, (d) rGO/Fe-doped TiO_2_, and (e) rGO/Ag/Fe-doped TiO_2_.

Fig. 5 shows the XRD patterns of commercially obtained graphite powder, and synthesized GO, rGO, rGO/Fe-doped TiO_2_, and rGO/Ag/Fe-doped TiO_2_ powders. In Fig. 5(a), graphite powder exhibits a sharp diffraction peak at 26.45°, corresponding to the inter-planar spacing of 0.33 nm and a somewhat less intense peak at 54.62°. These peaks can be attributed to the (002) and (004) hexagonal lattice planes of natural graphite, respectively. The diffraction pattern of GO in Fig. 5(b) shows a little broad peak at around 11.8°, corresponding to the (002) plane and the interlayer spacing of 0.75 nm, indicating the destruction of the graphite structure due to the oxidation and the structural conversion from graphite into GO.^43^ The XRD pattern of rGO is different from graphite and GO revealed a broader peak at 23.6° and 42.8°, corresponding to the reflection planes of (002) and (100) (Fig. 5(c)). The oxidation of graphite powder introduced numerous functional groups, which were bonded to the edges as well as both sides of the basal plane of the graphitic layer.^44^ For the synthesized rGO/Fe-doped TiO_2_ and rGO/Ag/Fe-doped TiO_2_ nanocomposite, the XRD patterns shown in Fig. 5(d) and (e) display several sharp peaks at 25.3, 37.8, 48.0, 53.9, and 62.7°, which are due to the (101), (004), (200), (105), and (204) planes for the anatase phase of TiO_2_ (JCPDS 21-1272), and a less-intense peak at 27.5° with (110) plane corresponding to the rutile phases (JCPDS78-1508), respectively. However, the metallic Ag phase cannot be detected in the synthesized photocatalysts due to a low loading amount of Ag. No iron oxide peaks were observed in the XRD pattern. It is presumed that the iron ions were totally incorporated into the structures of TiO_2_ and replaced titanium ions or were located at interstitial sites. On the basis of the (200) diffraction peak of rGO/Fe-doped TiO_2_, the estimated lattice parameters for *a*, *b* and *c* are about 3.782, 3.782 and 9.515 Å, respectively.^45^

### 3.2. Optical characterization

**Fig. 6:**
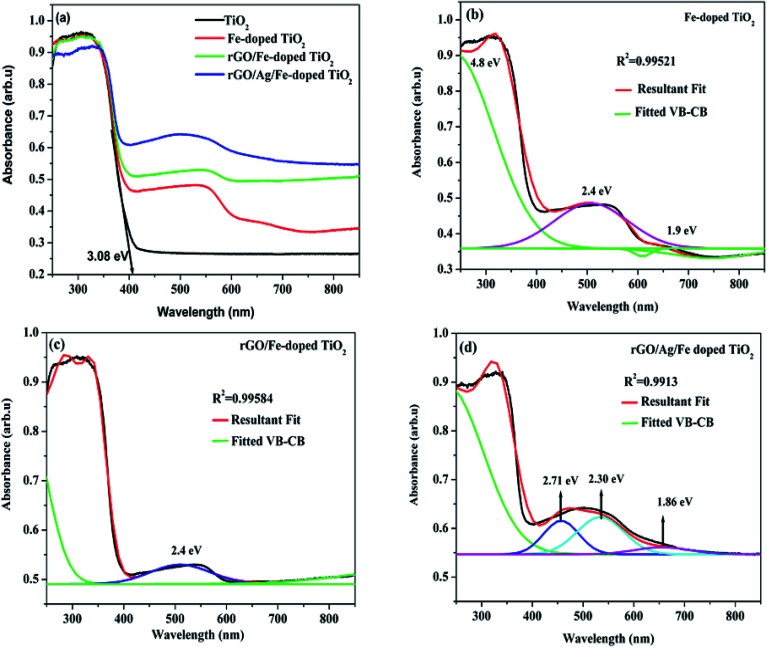
(a) Absorption spectra of TiO_2_, Fe-doped TiO_2_, rGO/Fe-doped TiO_2_, and rGO/Ag/Fe-doped TiO_2_. Deconvolution spectrum for (b) Fe-doped TiO_2_, (c) rGO/Fe-doped TiO_2_, and (d) rGO/Ag/Fe-doped TiO_2_.

Optical properties of the prepared samples were tested using UV-vis diffuse reflectance spectroscopy (DRS) (Fig. 6(a)). For undoped TiO_2_, the tangent line intercepts the *x*-axis at the wavelength of 402 nm and it corresponds to the bandgap energy of 3.08 eV. The red-shifts in the absorption edges were revealed for Fe-doped-, rGO/Fe-doped- and rGO/Ag/Fe-doped TiO_2_. When TiO_2_ is doped with Fe, the absorption edge spreads into the visible region, and the absorption edge corresponds to the electron transfer from the valence band (VB) to the conduction band (CB). Since Fe^3+^ in the 3d orbital is half filled, as Fe^3+^ is doped into TiO_2_, the empty E_g_ state is near the bottom of the conduction band, while the occupied t_2g_ state of Fe is located at the top of the valence band.^46^ There are multiple electronic transitions in Fe-doped TiO_2_, and there are multiple energy levels between the VB and CB. Fe-doped-, rGO/Fe-doped- and rGO/Ag/Fe-doped TiO_2_, showed broad absorption bands and d–d transitions of Fe^3+^ from the DRS spectra based on a deconvolution of the data (Fig. 6(b)–(d)). For Fe-doped TiO_2_, a strong transition observed near 4.8 eV corresponds to the charge transfer excitations of the 3d electrons of Fe^3+^ to the TiO_2_ CB and the broad absorption band from 400–700 nm assigned to the d–d transitions of Fe^3+^ (^2^T_2g_ → ^2^A_2g_, ^2^T_1g_) or to the charge transfer transition between interacting iron ions *via* the conduction band (Fe^3+^ + Fe^3+^ → Fe^4+^ + Fe^2+^).^11,47,48^ For rGO/Ag/Fe-doped TiO_2_, there is a broad range absorption in the visible region, mainly due to the local surface plasmonic resonance (LSPR) effect of Ag nanoparticles. This effect could enhance the solar-energy-conversion efficiency by increasing light absorption to longer wavelength and motivating photogenerated-carriers in the semiconductor by transferring the plasmonic energy from the Ag^0^ to the TiO_2_ semiconductor.^49^

**Fig. 7:**
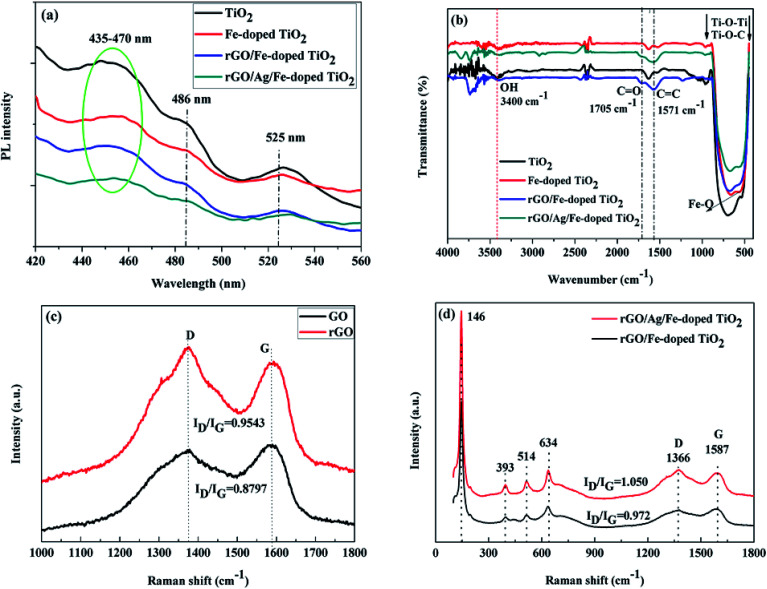
(a) PL emission spectra (b) FTIR spectra (c and d) Raman spectra of TiO_2_, Fe-doped TiO_2_, rGO/Fe-doped TiO_2_, and rGO/Ag/Fe-doped TiO_2_.

To further figure out the effect of rGO and Ag on the electron–hole separation for rGO/Ag/Fe-doped TiO_2_, photoluminescence spectroscopy (PL) was employed, particularly to characterize the recombination probability for photocatalysts. Fig. 7(a) shows that there is a broad emission band at around 435–470 nm, which could be assigned to the charge transfer transition of oxygen vacancy trapped electrons in TiO_2_.^50^ The excitonic PL signal at around 486 nm is related to the surface oxygen vacancies or defects in the specimen, and the PL signal located at the 525 nm band may originate from the F^+^ center on the surface of the TiO_2_.^51,52^ Notably, the luminescence intensities of the rGO/Ag/Fe-doped TiO_2_ were lower compared to other photocatalysts, which confirmed the lower electron–hole recombination probability for the rGO/Ag/Fe-doped TiO_2_ compared with others. This revealed that rGO and Ag nanoparticles slow down the electron–hole pair recombination in the photocatalyst, which may increase the photocatalytic activity.

The molecular structures of the TiO_2_, Fe-doped TiO_2_, rGO/Fe-doped TiO_2_ and rGO/Ag/Fe-doped TiO_2_ composites were characterized by FTIR spectroscopy (Fig. 7(b)). The peak around 3400 cm^−1^ is due to physically adsorbed water molecules (OH) on the specimens.^53^ As Fe was doped into TiO_2_, a new characteristic peak located at 575 cm^−1^ was attributed to the stretching vibration of the Fe–O bond.^46^ After rGO connected with Fe-doped TiO_2_-based specimens, several functional groups could be seen, such as the peak located at 1571 cm^−1^ corresponding to C=C aromatic bonding, and the lowering of the absorption peak at around 1705 cm^−1^ assigned to the C=O stretching accredited to rGO. Typically, Ti–O–Ti and Ti–O–C bonds can be seen at low frequency bands around 450 to 900 cm^−1^, and their shifting towards a higher wavenumber, compared with Fe-doped TiO_2_, indicates the chemical interaction of Fe-doped TiO_2_ with rGO. Based on the FTIR spectra, the intensity of the absorption bands was somewhat decreased for the rGO/Ag/Fe-doped TiO_2_ composite compared with the others. This indicates that the composite was covalently implanted over rGO sheets; the rGO sheets may shield the transmittance of infrared rays. A Raman study was carried out to further explain the chemical, structural, and vibrational bands of the TiO_2_-based composite. Fig. 7(c) shows the Raman spectra of GO and rGO using 532 nm laser irradiation. There are two sharp peaks located at 1341 and 1587 cm^−1^, which can be attributed to the disordered carbon (D-band) and graphitic carbon (G-band), respectively. The G-band with 1587 cm^−1^ has *E*_2g_ symmetry and it is due to the in-plane bond-stretching motion of pairs of C sp^2^ atoms. The D-band with 1341 cm^−1^ is a breathing mode of *A*_1g_ symmetry, and the intensity of the D-band is strictly connected to the presence of the six-fold aromatic ring.^54^ The ratios of D- and G-band intensities (*I*_D_/*I*_G_) for GO and rGO were 0.8797 and 0.9543, respectively. The *I*_D_/*I*_G_ ratio of rGO is greater compared to that of GO, indicating that the reduction process changed the structure of GO, and there was an increase in the number of smaller sp^2^ domains for rGO.^55^Fig. 7(d) shows the Raman spectra for rGO/Ag/Fe-doped TiO_2_ and rGO/Ag/TiO_2_ composites, which revealed almost the same pattern, except for the ratio of D- and G-band intensities (*I*_D_/*I*_G_). This exhibited specific vibration modes centered at 146 (E_g_), 393 (B_1g_), 514 (B_1g_ + A_1g_) and 634 cm^−1^ (E_g_), indicating the presence of the anatase phase of TiO_2_, which is consistent with the XRD results. Moreover, after loading Ag nanoparticles, the intensity of the Raman peaks of anatase TiO_2_ as well as the intensity ratio of the D- and G-band (*I*_D_/*I*_G_ = 1.050) significantly increased, as compared to that without Ag-loading (*I*_D_/*I*_G_ = 0.972). This could be related to the LSPR effect of Ag nanoparticles, which is in agreement with the DRS and PL studies for the rGO/Ag/Fe-doped TiO_2_ composite. An increase in the intensities of the Raman peaks was observed on anatase TiO_2_ and rGO, which indicated that Ag nanoparticles were deposited on the surface of TiO_2_ and well connected to the rGO layers. Overall, the addition of rGO and Ag nanoparticles increased the charge conduction and light absorption.

**Fig. 8:**
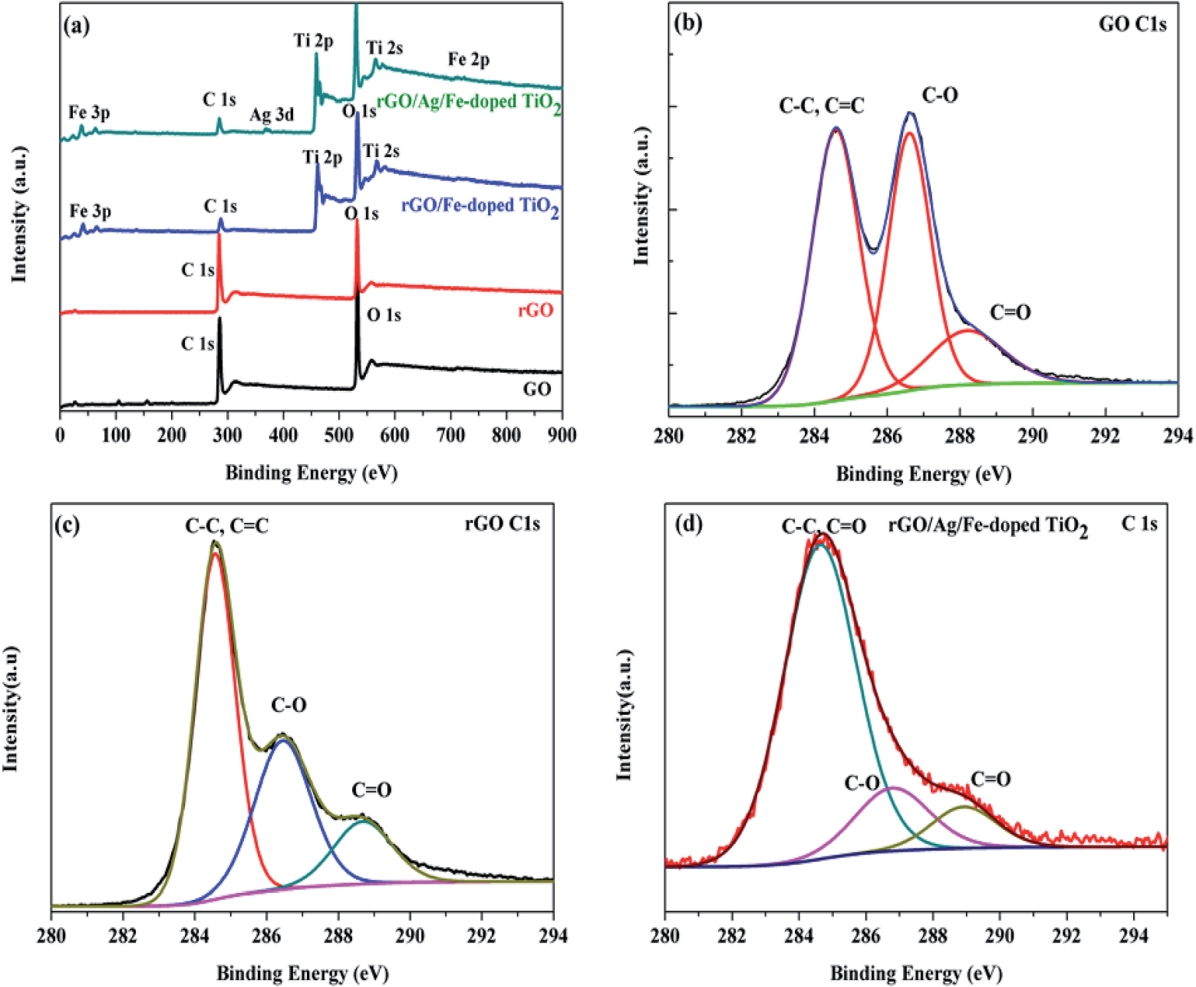
(a) XPS survey scan of GO, rGO, rGO/Fe-doped TiO_2_ and rGO/Ag/Fe-doped TiO_2_. (b) C 1s of GO, (c) C 1s of rGO, and (d) C 1s of rGO/Ag/Fe-doped TiO_2_ (inset shows the Ti 2p spectrum).

X-ray photoelectron spectroscopy (XPS) measurements were performed for comparison with the difference in the chemical state of carbon among GO, rGO, rGO/Fe-doped TiO_2_ and rGO/Ag/Fe-doped TiO_2_. Fig. 8(a) reveals the presence of C 1s, O 1s, Fe 2p, Ti 2p, and Ag 3d peaks in these materials. In Fig. 8(b) and (c), the XPS spectra of the GO and rGO for C 1s indicates that there are three peaks corresponding to the sp^2^ carbon at 284.6 eV for C=C/C–C, sp^3^ carbon at 286.6 eV for (C–O), and 288.4 eV for (C=O). After the reduction process, the peak intensity (C–O) for all the oxygen species decreased dramatically, suggesting the effective removal of the oxygen-containing groups in GO. In addition, a significant increase in the C=C/C–C peak indicated the restoration of the sp^2^ carbon network.^56^Fig. 8(d) shows that in the XPS spectrum of C 1s of the rGO/Ag/Fe-doped TiO_2_, the intensity of the peaks related to the oxygen functionalities became weaker than that of GO. The reduction in the peak intensities of the oxygen-functionalities declares the presence of residual oxygen-containing groups on rGO. It was observed that in the rGO/Ag/Fe-doped TiO_2_ composite, the C/O ratio was enhanced, which indicated that the rGO sheets can serve as a conductive channel between the metal oxide nanoparticles, and they are favorable for the photocatalytic process.^57^ The main broad peak was located at 284.6 eV and other less intense peaks were located at 286.6 and 288.4 eV, respectively. The presence of oxygenated weaker peaks indicates the deoxygenation of GO and formation of rGO. The spectrum in Fig. S1(a)† shows the Ti 2p-related peaks for rGO/Ag/Fe-doped TiO_2_, where the spin–orbit splitting of the Ti 2p peak leads to the doublets Ti 2p_3/2_ and Ti 2p_1/2_, which are located at 458.2 eV and 464.0 eV, respectively. The splitting of the 2p doublet is 5.8 eV, confirming the state of Ti^4+^ in the rGO/Ag/Fe-doped TiO_2_. Fig. S1(b)† shows the XPS spectra of Ag 3d_5/2_ and Ag 3d_3/2_ and the splitting of the 3d doublet is 6.1 eV, indicating the metallic nature of silver.^58,59^ Fig. S1(c)† shows the O 1s spectral peak at 531.3 eV for the surface O–H group. In Fig. S2,† XPS peaks were seen at 457.67 eV and 463.36 eV for Ti 2p_3/2_ and Ti 2p_1/2_, respectively, for the TiO_2_ specimen. In Fig. S3,† for the Fe^3+^ doped TiO_2_, two peaks located at 458.31 eV and 464.03 eV slightly shifted toward higher binding energy compared to TiO_2_, which confirmed the presence of the Fe^3+^ ions in Fe-doped TiO_2_. The Fe 2p XPS peaks located at 709.16 eV and 723.78 eV are attributed to the binding energies of the Fe 2p_3/2_ and Fe 2p_1/2_, also confirming the presence of the Fe^3+^ dopant in the TiO_2_ lattice. From Fig. 8(a) showing the survey spectrum of rGO/Ag/Fe-doped TiO_2_, the Ti 2p_3/2_ and Ti 2p_1/2_ peaks are located at 459.14 eV and 464.62 eV, and slightly shift towards higher binding energy compared to those of Fe-doped TiO_2_, which implies the interactions of Ti with the oxygen centers of rGO.

### 3.3. Photocatalytic activity and mechanism

**Fig. 9:**
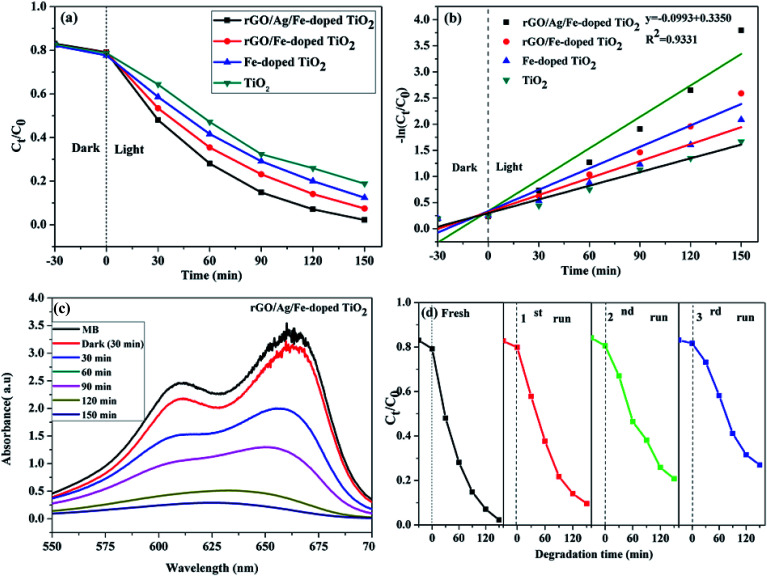
(a) Photodegradation of MB solution for four different photocatalysts. (b) Kinetics curves of the photocatalytic degradation. (c) Time-dependent UV-visible absorption spectra of the MB solution in the presence of rGO/Ag/Fe-doped TiO_2_. (d) Recycle testing of MB solution for the rGO/Ag/Fe-doped TiO_2_ composite.

**Fig. 10:**
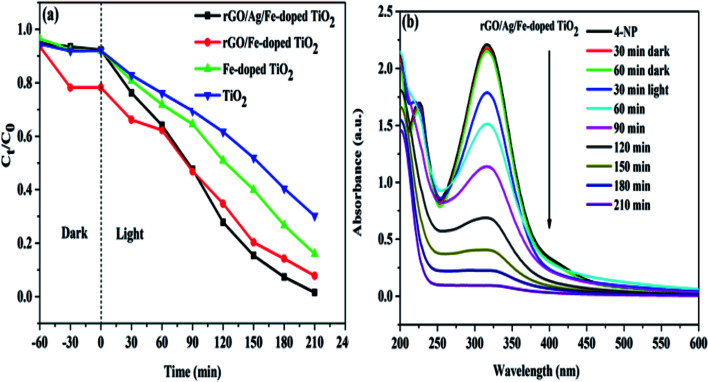
(a) Photodegradation of 4-NP solution for four different photocatalysts. (b) Time-dependent UV-visible absorption spectra of the 4-NP solution in the presence of rGO/Ag/Fe-doped TiO_2_.

The photocatalytic performance was tested in the photodegradation of MB (20 ppm) under irradiation with a 35 W Xe arc lamp, in aqueous solution and ambient conditions. The photodegradation efficiencies were 95.33, 88.79, 82.40, and 74.59% for rGO/Ag/Fe-doped TiO_2_, rGO/Fe-doped TiO_2_, Fe-doped TiO_2_, and pure TiO_2_, respectively, within 150 min of irradiation (Fig. 9(a)). It was assumed that the degradation of the MB solution under visible light obeyed the pseudo-first order reaction kinetics as follows:1
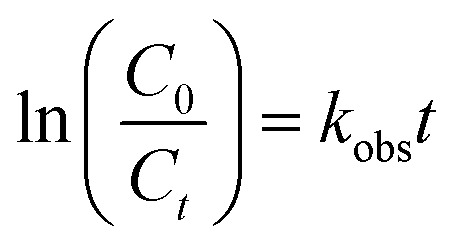
where *C*_0_ is the initial concentration, *C*_*t*_ is the concentration at time (*t*), and *k*_obs_ is the apparent rate constant (time^−1^). Based on eqn (1), the plot of ln(*C*_0_/*C*_*t*_) *versus* illumination time (*t*) represents a straight line, and the slope of linear regression is equal to the apparent first-order rate constant, *k*_obs_. The values for the degradation rate constant are 0.020, 0.014, 0.011, and 0.0087 min^−1^ for rGO/Ag/Fe-doped TiO_2_, rGO/Fe-doped TiO_2_, Fe-doped TiO_2_, and TiO_2_, respectively (Fig. 9(b)). The absorption spectra of the MB solution in the presence of the rGO/Ag/Fe-doped TiO_2_ composite for different illumination times are shown in Fig. 9(c). In addition to the degradation activity of the photocatalysts, their stability is also significant for their practical applications. The rGO/Ag/Fe-doped TiO_2_ composite with highest photocatalytic property was selected for the recycling degradation experiment (Fig. 9(d)). The degradation percentages for the three cycles were 87, 76, and 69%, respectively. The effect of the degradation of different MB concentrations with constant rGO/Ag/Fe-doped TiO_2_ (10 mg) was investigated and the error bar diagram with the standard deviation is shown in Fig. S4.† Also, the degradation of other types of organic pollutant such as 4-NP was tested in the presence of photocatalysts as shown in Fig. 10. The photodegradation efficiencies were 95.66, 87.17, 80.35, and 65% for rGO/Ag/Fe-doped TiO_2_, rGO/Fe-doped TiO_2_, Fe-doped TiO_2_, and pure TiO_2_, respectively, within 210 min irradiation (Fig. 10(a)). The absorption spectra of the 4-NP solution in the presence of the rGO/Ag/Fe-doped TiO_2_ composite for different illumination times is shown in Fig. 10(b).

**Fig. 11:**
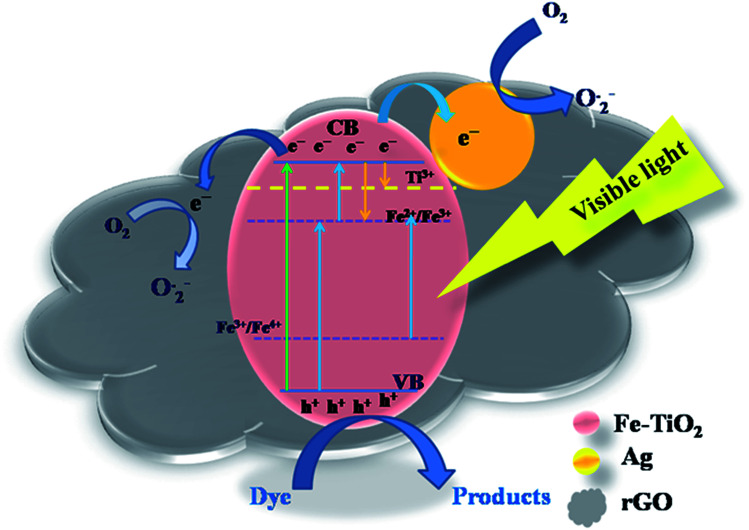
Schematic of the mechanism of charge separation for the rGO/Ag/Fe-doped TiO_2_ under the 35 W Xe arc lamp.

Based on the experimental data discussed above, we propose a possible photocatalysis mechanism in Fig. 11. Generally, the photogenerated electrons in the conduction band are consumed by the surface adsorbed oxygen molecules to produce superoxide anion (O_2_^−^˙) radicals, while generated holes in the valence band are scavenged by surface hydroxyl groups to generate hydroxyl radicals (OH˙). Subsequently, these radicals can be used for the degradation of the pollutant. UV-vis spectra revealed that the doping of Fe^3+^ ions in the TiO_2_ lattice modified its original band structure. The visible-light response for the Fe-doped TiO_2_ specimen is due to narrowing of the band gap, which is attributed to the following: (1) the d–d transition between Fe^3+^ ions and conduction band electrons; (2) charge transfer transitions between interacting Fe^3+^ ions to create electronic states (Fe^4+^ and Fe^2+^) that are spread across the band gap of TiO_2_. These different electronic states act as electron- and hole-trapping sites. They decrease the electron–hole pair recombination rate and enhance photocatalytic activity. Fe^3+^ can act as a hole trapper (Fe^3+^ + h^+^ → Fe^4+^) because the energy level of Fe^3+^/Fe^4+^ lies above the valence band. The trapped holes in Fe^4+^ can migrate to the surface adsorbed hydroxyl ions to produce hydroxyl radicals (Fe^4+^ + OH^−^ → Fe^3+^ + OH˙). Fe^3+^ ions can also serve as trapping sites for both photogenerated electrons and the electrons relaxed from the conduction band. They form Fe^2+^ ions *via* the reduction reaction (Fe^3+^ + e^−^ → Fe^2+^). These Fe^2+^ ions are oxidized to Fe^3+^ ions by transferring the electrons to the adsorbed O_2_ molecules on the catalyst surface (Fe^2+^ + O_2ads_ → Fe^3+^ + O_2_^−^˙).^60^ The O_2_^−^˙ ions can easily trap photogenerated holes, and hydroxyl radicals and hydroxyl ions are produced (O_2_^−^˙ + h^+^ → O^−^˙; O^−^˙ + H_2_O_ads_ → + OH˙ + OH^−^).^48^ Because the Fe^2+^/Fe^3+^ energy level is close to that of Ti^3+^/Ti^4+^, it is possible that the trapped electrons in Fe^2+^ are transferred to Ti^4+^, and anion radicals are generated upon reacting with adsorbed oxygen (Fe^2+^ + Ti^4+^ → Ti^3+^ + Fe^3+^; Ti^3+^ + O_2_ → O_2_^−^˙ + Ti^4+^).^61^ However, as the Fe^3+^ doping-level exceeds a certain amount due to a decrease in the distance between trapping sites, Fe^3+^ ions may also act as the recombination centers of the photogenerated electrons and holes, which is unfavorable to photocatalytic reaction. To overcome this problem, metal Ag^0^ particles are deposited on the catalyst surface and can act as electron traps. Since the Schottky barrier is formed between the Ag NPs and Fe-doped TiO_2_ interface, the electrons migrate from Fe-doped TiO_2_ to the Ag NPs.^50^ Simultaneously, the LSPR effect is induced under visible light irradiation and produces a strong local electronic field that enhances the energy of trapped electrons. The Fermi level of rGO is below the potential of the conduction band of TiO_2_, and it is speculated that rGO may act as an electronic acceptor that could accept electrons from the valence band. Thus, both Ag and rGO could serve as acceptors for the photogenerated electrons from the valence band of Fe-doped TiO_2_.

**Fig. 12:**
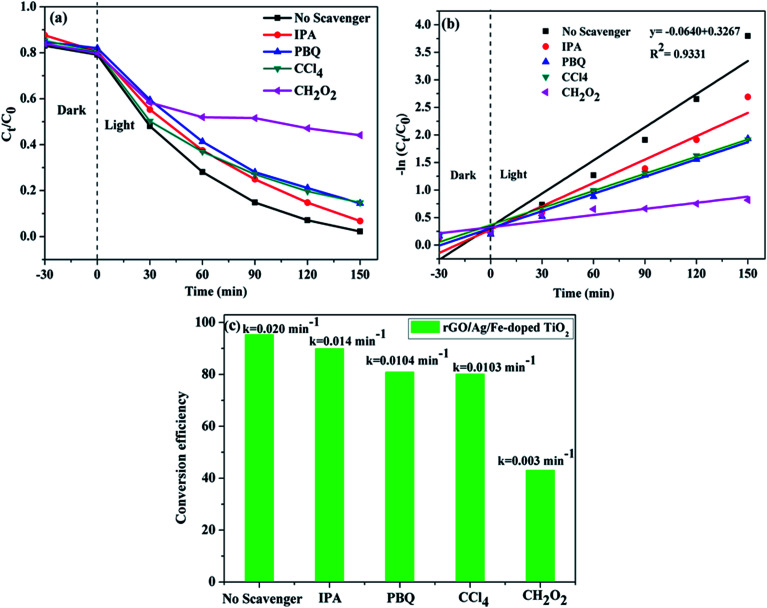
(a) Photocatalytic degradation of MB in the presence of scavengers over the rGO/Ag/Fe-doped TiO_2_ composite. (b) First order kinetic curves of MB degradation with different kinds of scavengers. (c) Degradation conversion efficiency for different scavengers.

To further understand the photocatalytic mechanism, it is important to detect the main oxidative species of the photocatalytic process for explaining the photocatalytic mechanism. The main oxidative species in the photocatalytic process were detected through the radicals-trapping experiment using IPA as the hydroxyl radical scavenger and CH_2_O_2_ as a hole scavenger. Tetrachloromethane (CCl_4_) and *para*-benzoquinone (PBQ) were used as the electron and superoxide radical scavengers.^61,65^Fig. 12(a) reveals the photocatalytic degradation of MB addition with various scavengers for the rGO/Ag/Fe-doped TiO_2_ composite. Fig. 12(c) shows that in the presence of CH_2_O_2_, a scavenger of holes, the degradation conversion efficiency is only 43%, indicating that the photogenerated holes (h^+^) accumulated in the valence band of Fe-doped TiO_2_; they are one of the main reactive species for the degradation of MB that could directly oxidize the pollutants. On the contrary, the degradation conversion efficiency is close to 90% in the presence of IPA, a scavenger of hydroxyl radicals (OH˙), indicating that the hydroxyl radicals are not the main reactive species for MB degradation. In the presence of PBQ and CCl_4_ for scavengers of photogenerated electrons (e^−^) and superoxide radicals (O_2_^−^˙), they revealed the minor reactive species for MB degradation. It was concluded that the most important radicals to degrade MB solution are ranked as follows: h^+^ > O_2_^−^˙ > e^−^ > OH˙. The first order kinetic plots for MB degradation for different scavengers are shown in Fig. 12(b). Fig. 12(c) addresses the values of rate constants. Table 1 lists the comparison of composite photocatalysts and their photocatalytic performances.

**Table 1:** Comparison of composite photocatalysts and their photocatalytic performances

Material	Pollutant & catalyst loading	Light source	Degradation (%)	Degradation time (min)/temperature	Stability performance (no. of recycle tests)	Ref.
Graphene/Fe–TiO_2_ nanowire	MB (10 ppm) & 100 mg	400 W Xe lamp	99.5	80 min/room temp.	5 cycles	62
Fe_3_O_4_@rGO@TiO_2_	MB (10 ppm) & 0.1 to 1.5 mg L^−1^	300 W UV-vis lamp	99	140/room temp.	6 cycles	63
TiO_2_–Ag/GR	MB (1 ppm–5 ppm) & 1 mg L^−1^ −5 mg L^−1^	HP Hg lamp	100	160/pH-6/room temp.	5 cycles	64
TiO_2_@C/Ag	RB and MO (5 ppm) & 0.03 g	150 W Xe lamp	91 and MO not mentioned	360 min/room temperature	3 cycles	59
rGO-Fe_3_O_4_–TiO_2_	MB (1 mg L^−1^) & 0.5 mg mL^−1^	125 W HPMV lamp	100% under UV light & 91% in visible light	5 min/room temp.	3 cycles	7
Ag–Cu_2_O/rGO composite	MO (32 mg L^−1^) & 10 mg	400 W metal halide lamp	95%	60 min/room temp.	3 cycles	56
TiO_2_-rGO composite	MB (10 ppm) & 1 mg mL^−1^	LED torches (∼0.1 mW mm^−2^)	98.72	300 min/room temp.	—	60
Fe doped TiO_2_	MB (7.5 ppm) & 100 mg L^−1^	UV light	>95	60 min/room temp.	6 cycles	11
rGO/Ag/Fe doped TiO_2_	MB (20 ppm) & 10 mg	35 W Xe arc lamp	95.33	150 min/room temp.	4 cycles	This work

### 3.4. Photoelectrochemical properties

**Fig. 13:**
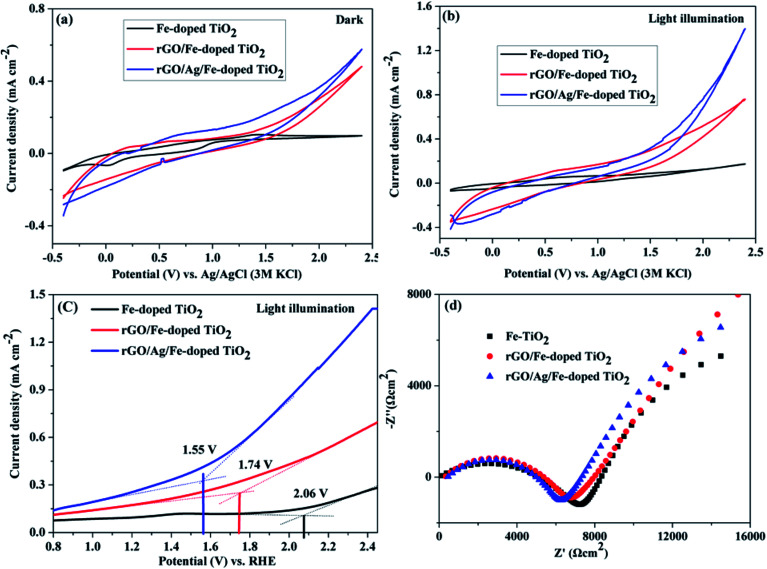
Cyclic voltammogram of Fe-doped TiO_2_, rGO/Fe-doped TiO_2_, and rGO/Ag/Fe-doped TiO_2_ in (a) dark and (b) 35 W light illumination in 1 M H_2_SO_4_ at 50 mV s^−1^ scan rate (V *vs.* Ag/AgCl). (c) LSV under 35 W light illumination (V *vs.* RHE) and (d) EIS spectra.

The photoelectrochemical properties of Fe-doped TiO_2_, rGO/Fe-doped TiO_2_ and rGO/Ag/Fe-doped TiO_2_ were studied in 1 M H_2_SO_4_ electrolyte. Fig. 13(a) and (b) show cyclic voltammograms (under dark conditions and under 35 W light irradiation in 1 M H_2_SO_4_ solutions). The current densities were observed at the anodic vertex of 2.4 V for dark and light irradiation; rGO/Ag/Fe-doped TiO_2_ has the highest current density of 1.39 mA cm^−2^ under the light. Table S1† shows the current densities of all materials under dark and light conditions. Fig. 13(c) shows linear sweep voltammetry curves for the photocatalysts with light irradiation and EIS spectra, respectively. The oxygen evolution reaction (OER) begins as an onset potential of 2.06 V for Fe-doped TiO_2_ (*η* = 0.83 V), 1.74 V for rGO/Fe-doped TiO_2_ (*η* = 0.51 V), and 1.55 V for rGO/Ag/Fe-doped TiO_2_ (*η* = 0.32 V) with respect to the reversible hydrogen electrode (RHE), where *η* is the overpotential. The onset potential of OER for rGO/Ag/Fe-doped TiO_2_ (1.55 V *vs.* RHE) is a little higher than for the in-house synthesized as well as commercially obtained IrO_2_ (onset potential 1.43 V *vs.* RHE).^66^ The rGO/Ag/Fe-doped TiO_2_ has a low overpotential compared to Fe-doped TiO_2_. This is one of the best attributes of our photocatalysts and further investigation is needed to improve the electrochemical activity of metal oxide based photocatalysts for OER activity. Fig. 13(d) reveals the Nyquist plot; the electron transfer resistance (*R*_et_) for rGO/Ag/Fe-doped TiO_2_ is about 6 kΩ cm^−2^, for rGO/Fe-doped TiO_2_ it is about 7 kΩ cm^−2^, and for Fe-doped TiO_2_ it is about 8 kΩ cm^−2^. Fig. S5† shows the corresponding equivalent circuit. The results suggest that the rGO/Ag/Fe-doped TiO_2_ is a good photocatalyst for maintaining OER activity.

## 4. Conclusion

In summary, 1 wt% of Ag nanoparticles loaded Fe-doped TiO_2_ on rGO (rGO/Ag/Fe-doped TiO_2_) was successfully prepared and photocatalytic MB degradation was examined under a 35 W Xe arc lamp. The rGO/Ag/Fe-doped TiO_2_ revealed the highest MB solution degraded performance for which photocatalytic conversion efficiency reached 95.33% in 150 min, and the rate of degradation constant *k* was 0.020 min^−1^. The DRS study used trap levels and band gap tuning in TiO_2_ to investigate the photocatalytic activity in near-visible wavelength conditions. The photocatalytic mechanism was evaluated by using different kinds of radical scavengers. The photoelectrochemical studies of OER were carried out, and the overpotential *η* was 0.32 V for rGO/Ag/Fe-doped TiO_2_ and the corresponding current density was 1.39 mA cm^−2^ under the light illumination. Overall, the studies suggest that the rGO/Ag/Fe-doped TiO_2_ has a good photocatalytic activity towards MB degradation and maintains OER activity in an acid electrolyte.

## Conflicts of interest

There are no conflicts of interest to declare.

## Supplementary Material

RA-008-C7RA13418E-s001
