# Supplementary material for: Reduced graphene oxide-supported Ag-loaded Fe-doped TiO2 for the degradation mechanism of methylene blue and its electrochemical properties
Source: RSC Adv. 2018 Feb 9;8(12):6488–501. doi: 10.1039/c7ra13418e (PMC9078252; doi:10.1039/c7ra13418e)
Supplement: RA-008-C7RA13418E-s001 [file RA-008-C7RA13418E-s001.pdf]

Electronic Supplementary Material (ESI) for RSC Advances.
This journal is © The Royal Society of Chemistry 2018

# Electronic Supplementary Information

## Reduced graphene oxide supported Ag-loaded Fe-doped $TiO_2$ for methylene blue degradation mechanism and its electrochemical properties

Dhayanantha Prabu Jaihindh, Ching Cheng Chen, Yen Pei Fu*
Department of Materials Science and Engineering, National Dong Hwa University, Shou-Feng, Hualine, 97401, Taiwan
* Corresponding author: ypfu@gms.ndhu.edu.tw

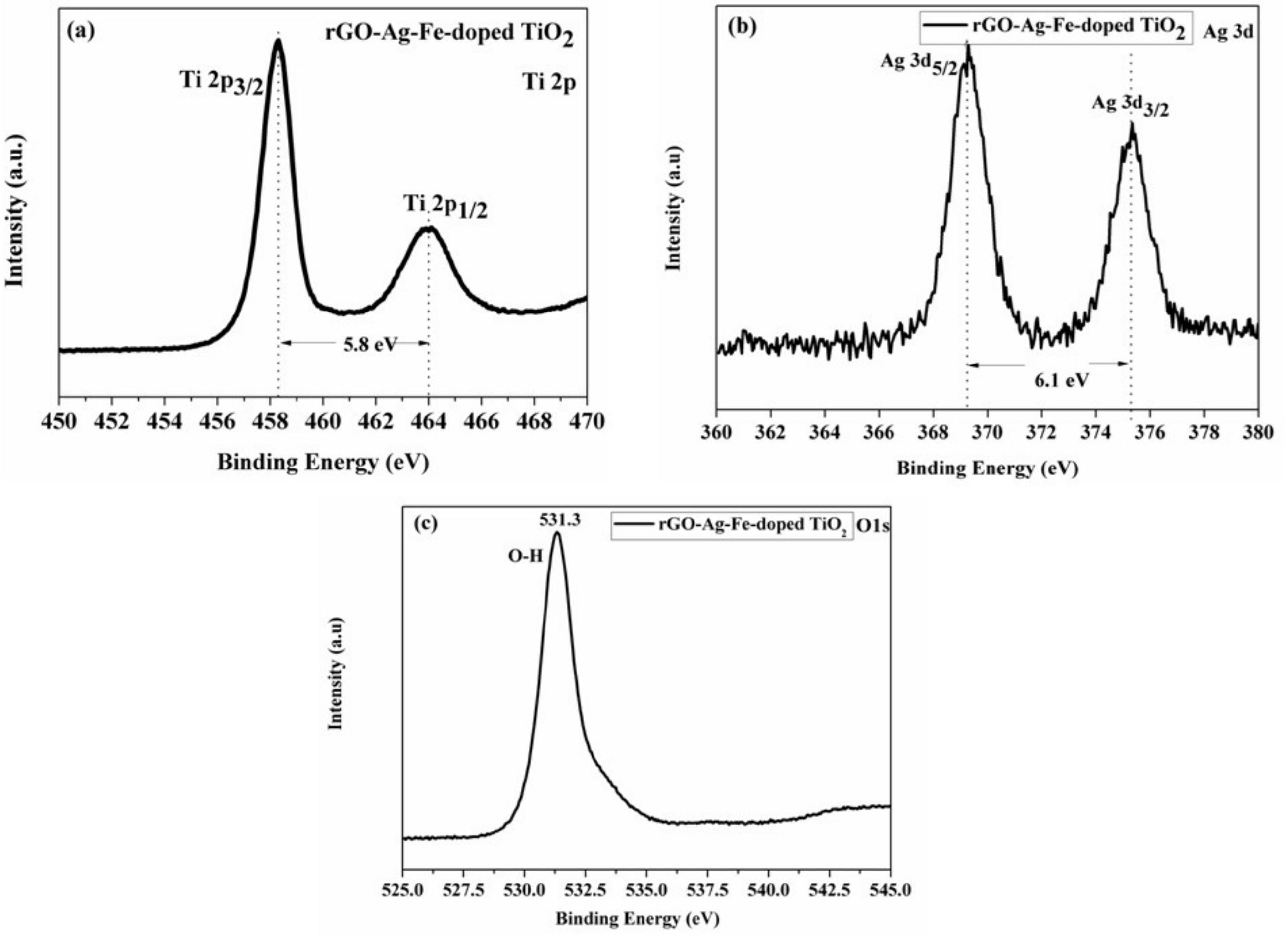


**Figure S1.** (a) Ti 2p, (b) Ag 3d, and (c) O 1s spectrum for rGO/Ag/Fe-doped $TiO_2$.

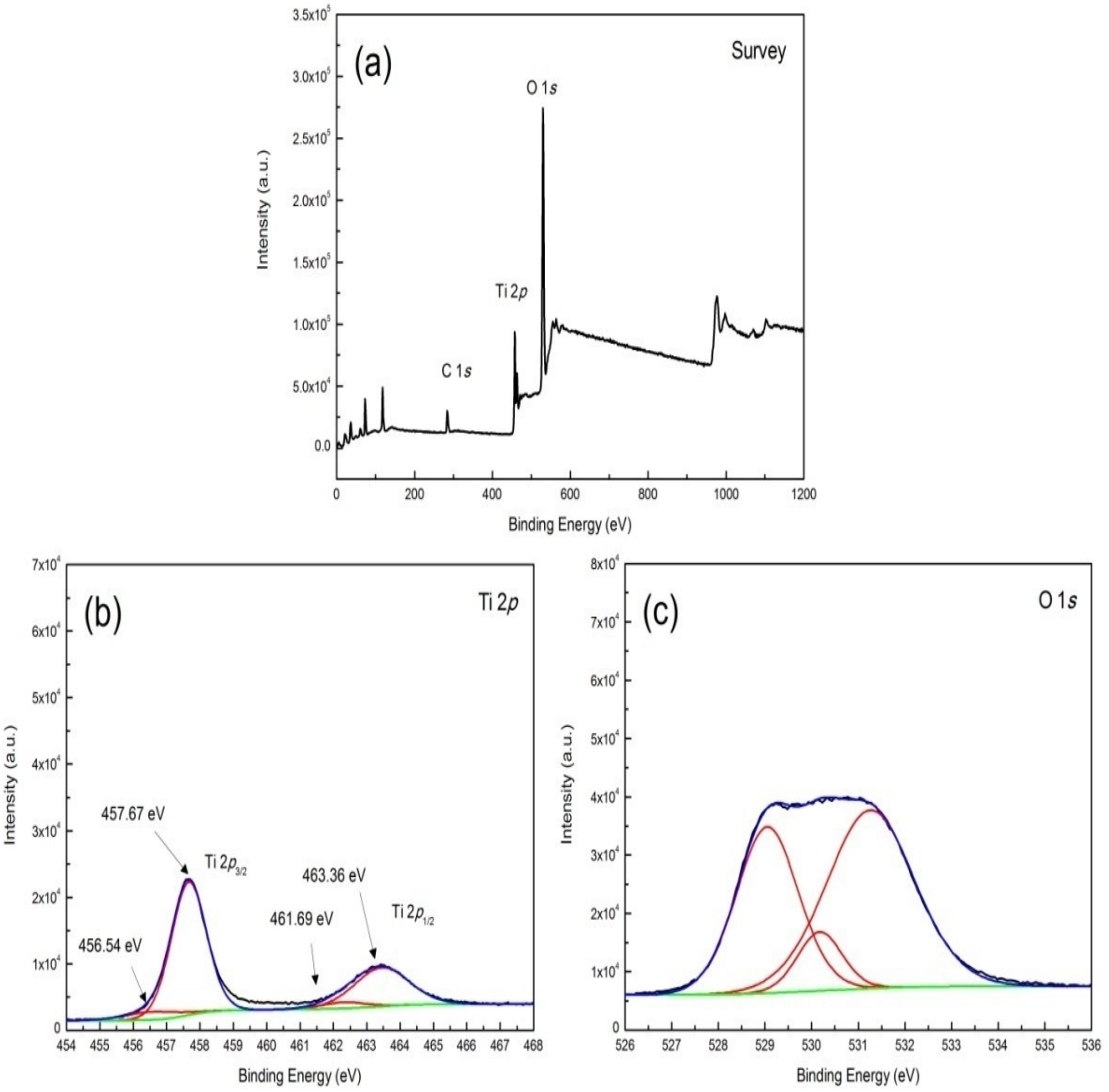


**Figure S2. XPS spectra of $TiO_2$**

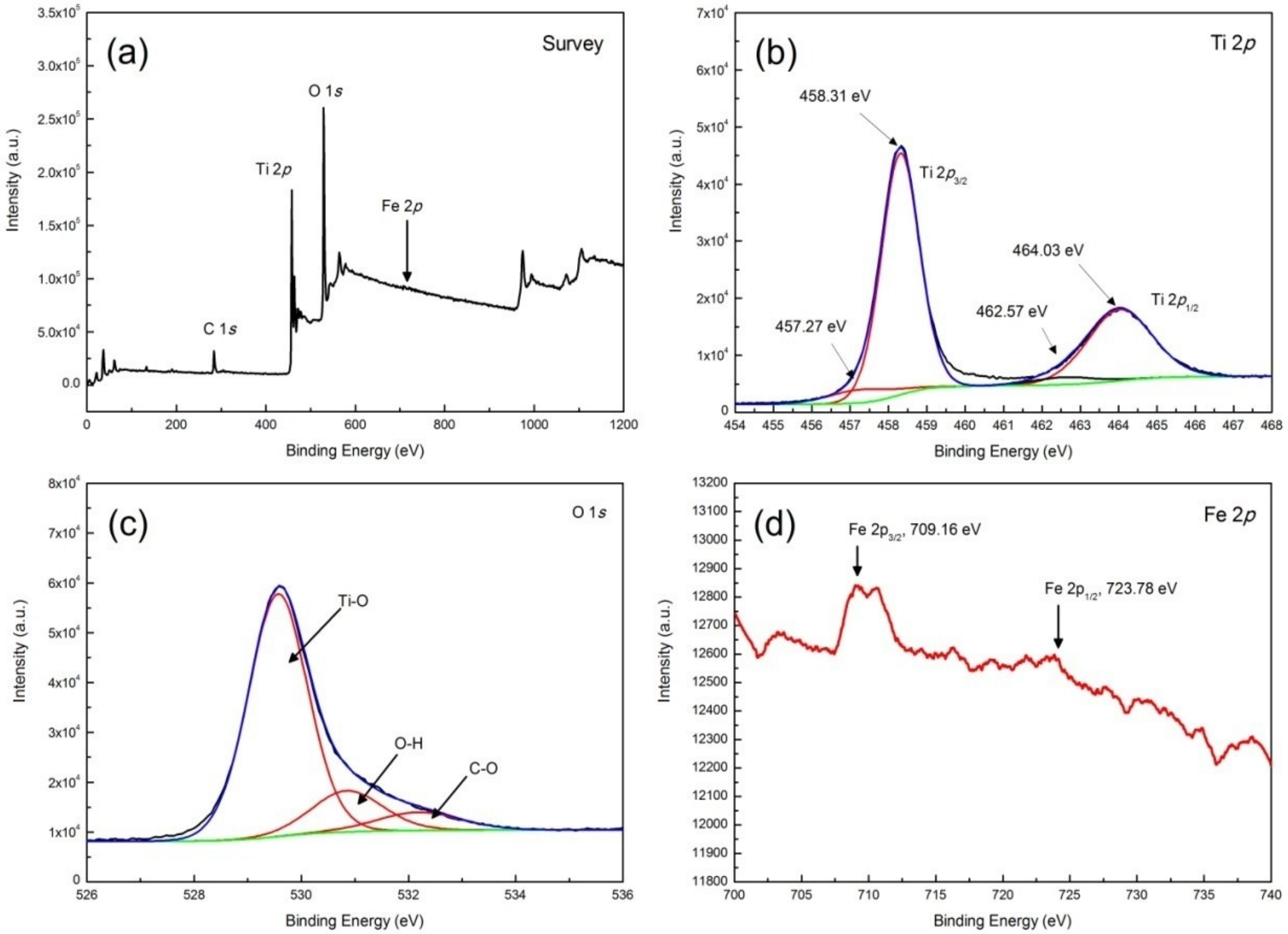


**Figure S3. XPS spectra of Fe-doped $TiO_2$**

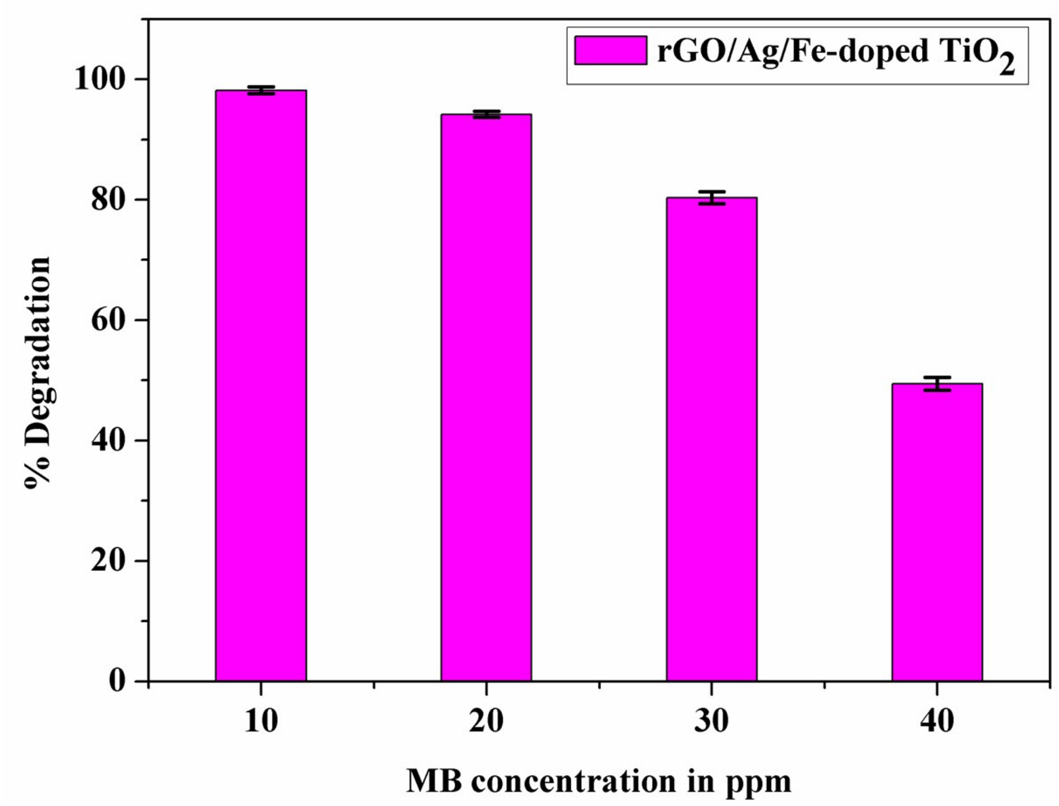


**Figure S4.** Effect of different MB concentration on rGO/Ag/Fe-doped $TiO_2$ (10 mg). Error bars represent standard deviations of three-times measurements

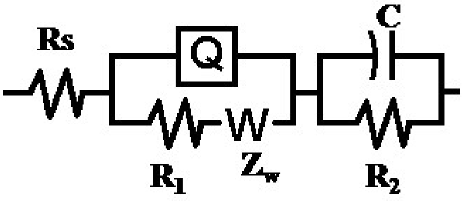


**Figure S5:** Equivalent circuit of EIS

Rs: Solution resistance

Q: Constant phase element Q=C (capacitor)

$Z_W$: Warburg constant

$R_1$,$R_2$: Charge transfer resistance

**Table S1:** Current density values in dark and light irradiation

| Photocatalysts | Current density (mA $cm^{-2}$) In dark | Current density (mA $cm^{-2}$) In light |
|---|---|---|
| Fe-doped $TiO_2$ | 0.102 | 0.176 |
| rGO/Fe-doped $TiO_2$ | 0.473 | 0.755 |
| rGO/Ag/Fe-doped $TiO_2$ | 0.571 | 1.39 |
